# Supplementary material for: A scalable filtration-based method for isolating exomeres and other nanoscale extracellular particles
Source: bioRxiv. 2025 Oct 24:2025.10.23.684032. Preprint. [Version 1] doi: 10.1101/2025.10.23.684032 (PMC12633226; doi:10.1101/2025.10.23.684032)
Supplement: Supplement 1 [file media-1.pdf]

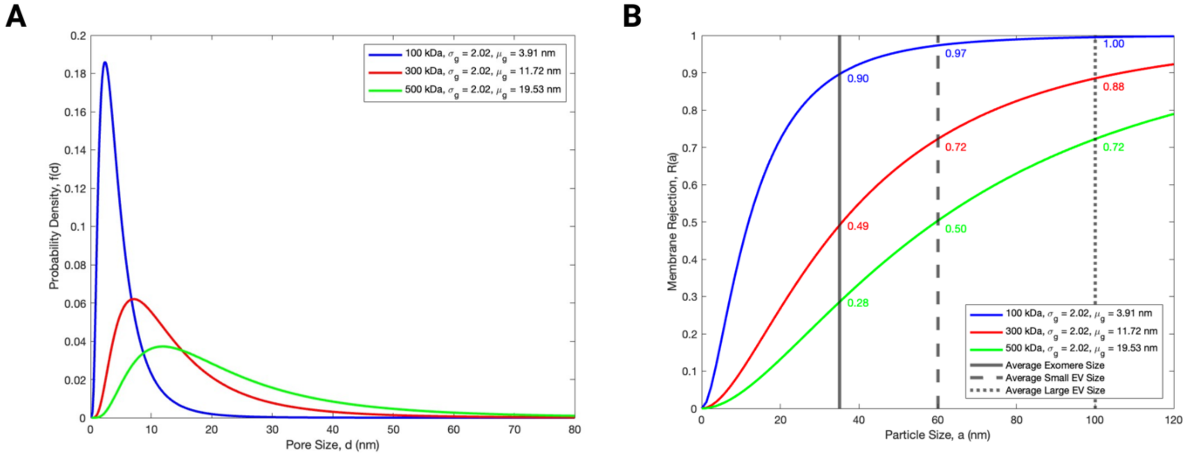

Supplementary Figure 1: Filter rejection predictions using log-normal models previously described.[1] **A.** Probability density functions showing the theoretical pore size distribution for 100, 300, and 500 kDa MWCO membranes. **B.** Membrane rejection performance for particles with a size of 35 nm (exomeres), 60 nm (sEVs), and 100 nm (LEVs). The value chosen for geometric standard deviation (σ_g) was adopted from prior literature based on measurements of asymmetric, modified polyethersulfone (PES) membranes which well-represent the commercial membranes used in this study.[2] Geometric means (μ_g) were calculated from σ_g and the mean pore diameter reported by the manufacturer.

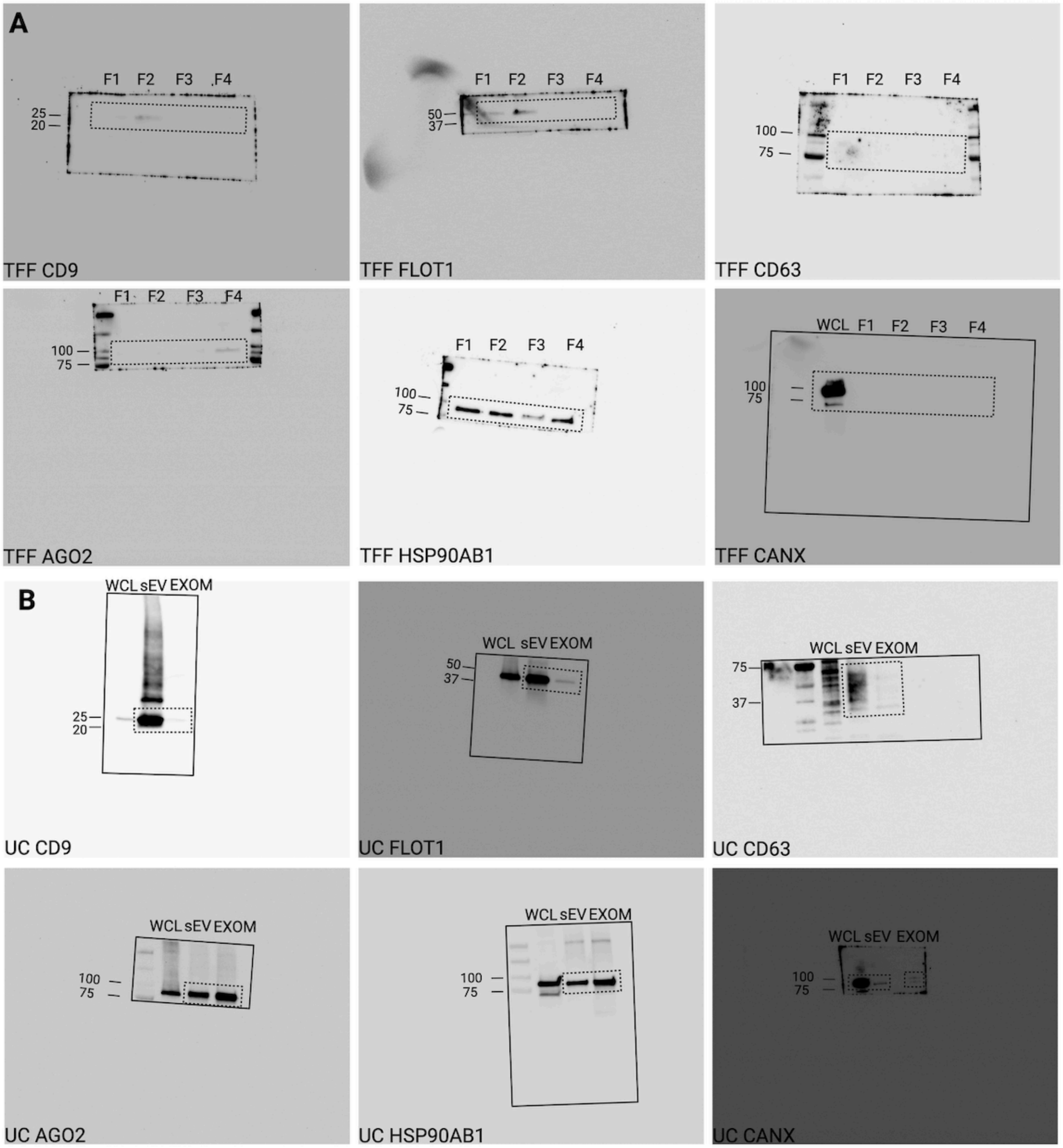

Supplementary Figure 2: Unprocessed Western blots for **A.** Tandem TFF and **B.** Differential UC. Dotted boxes represent the regions of the blots presented in **Fig. 2**. Solid boxes represent the outline of the complete blot.

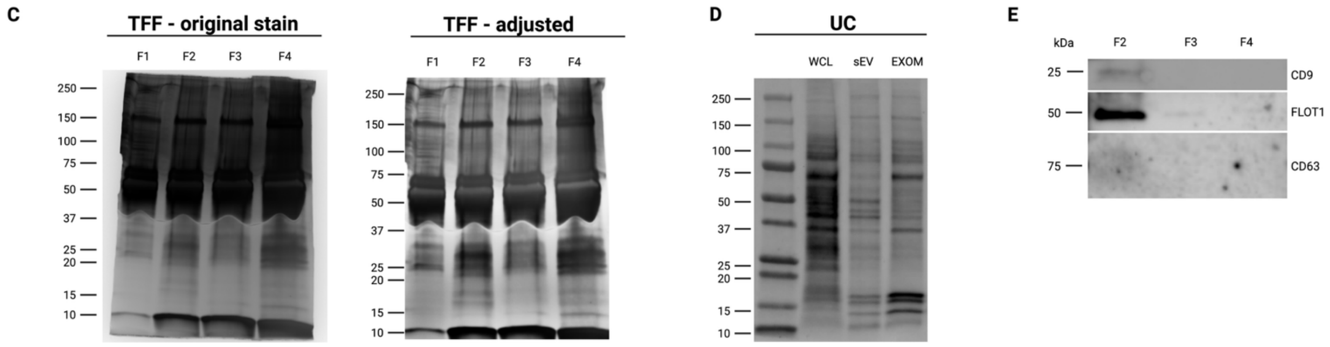

Supplementary Figure 2 (continued): Total protein visualization by **C**. silver staining for TFF fractions and **D.** Revert700 fluorescent staining for UC fractions. **E.** Femto ECL Western blot showing absence of EV markers in NVEP fractions F3 and F4 in comparison to EVs in F2.

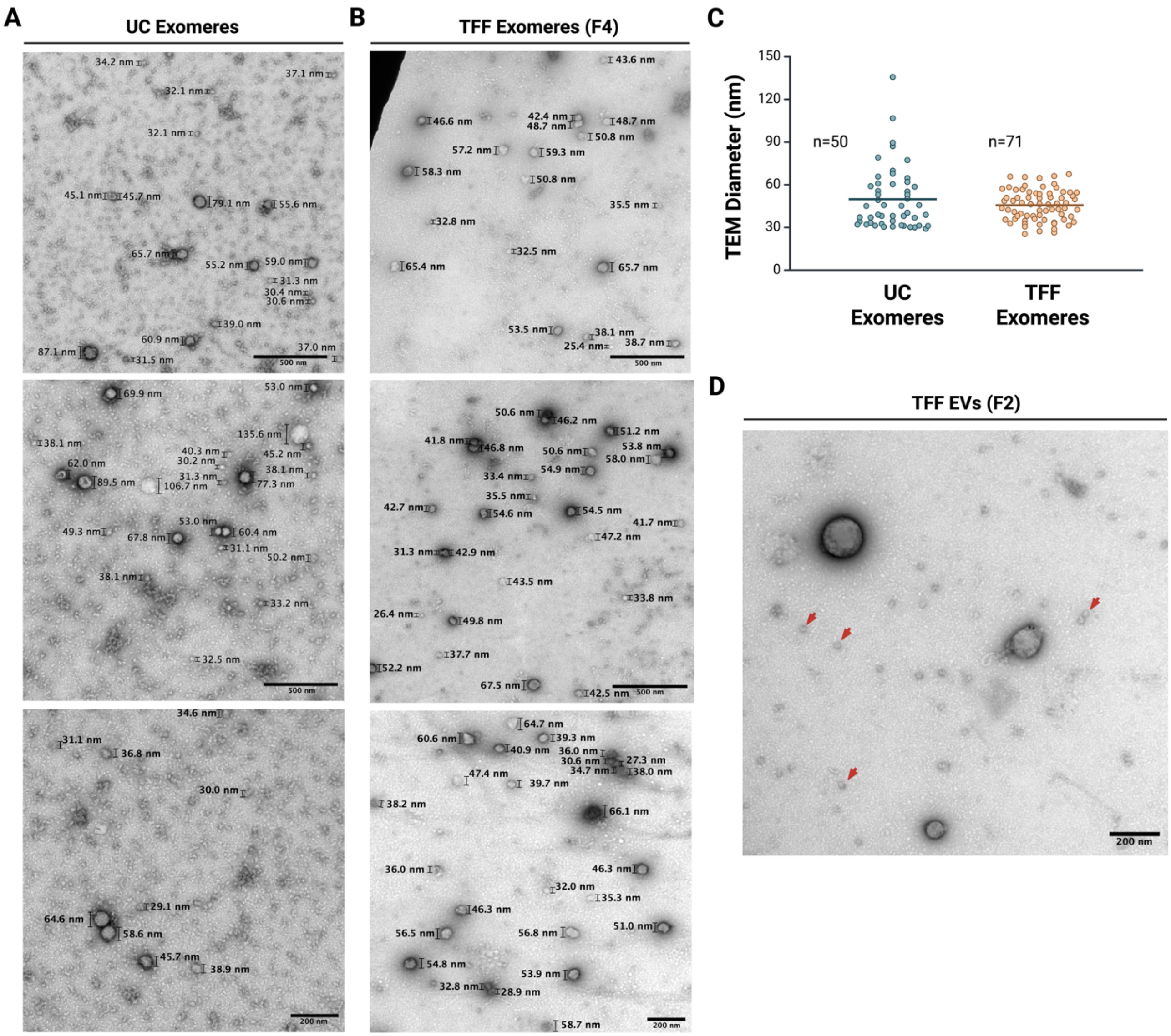

Supplementary Figure 3: TEM micrographs of particles measured for **A.** UC exomeres and **B.** TFF exomeres (F4). **C.** Size distribution of particles within each fraction based on manual measurement using ImageJ 1.54g (Java 1.8.0_345). **D.** Exomere-shaped particles co-purify in TFF EV fraction F2. Figure created with BioRender.com.

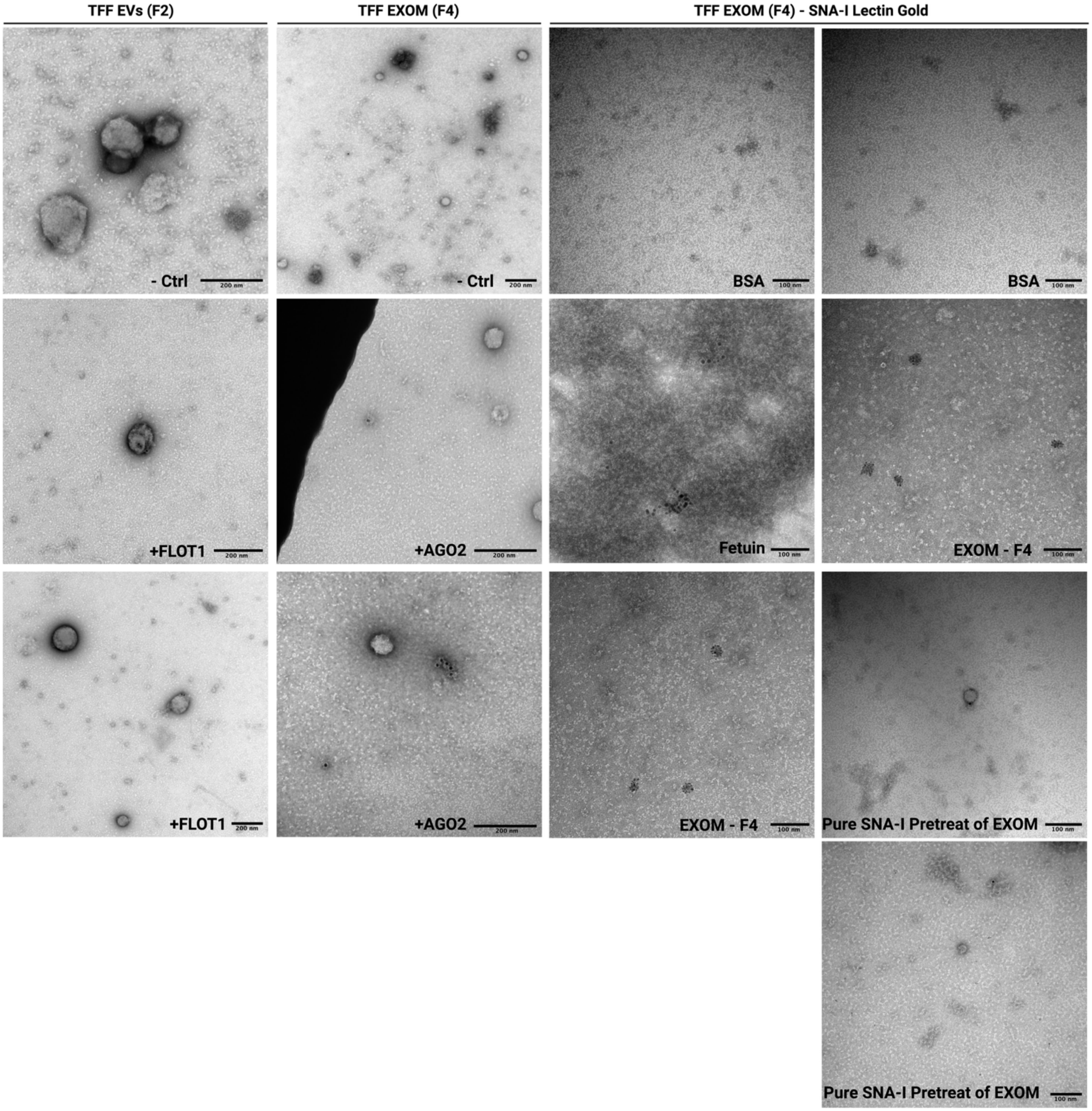

Supplementary Figure 4: Unprocessed TEM micrographs for immunogold and SNA-I lectin gold experiments.

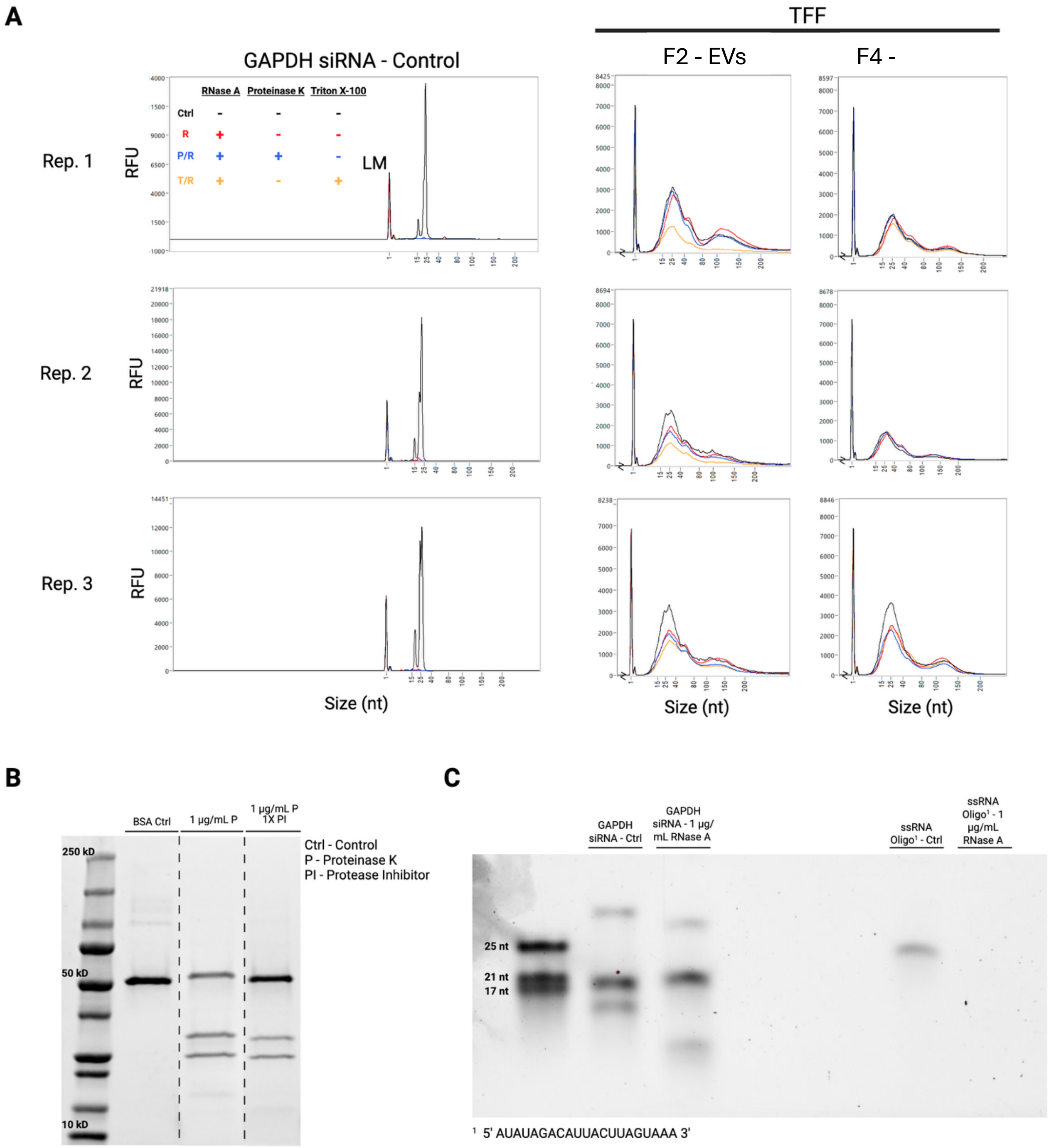

1 5' AUAUAGACAUUACUUAGUAAA 3'

Supplementary Figure 5: A. RNA protection assay results representing three biological replicates. Treatment conditions: Ctrl (Control), R (RNase A only), P/R (Proteinase K/RNase A), T/R (Triton X-100/RNase A). **B**. Coomassie blue-stained PAGE gel of BSA treated with Proteinase K or Proteinase K and inhibitor. Gel was cropped to remove conditions not used in the RNase protection experiment. **C.** SYBR^{TM} gold-stained PAGE gel of dsRNA and ssRNA treated with RNase A.

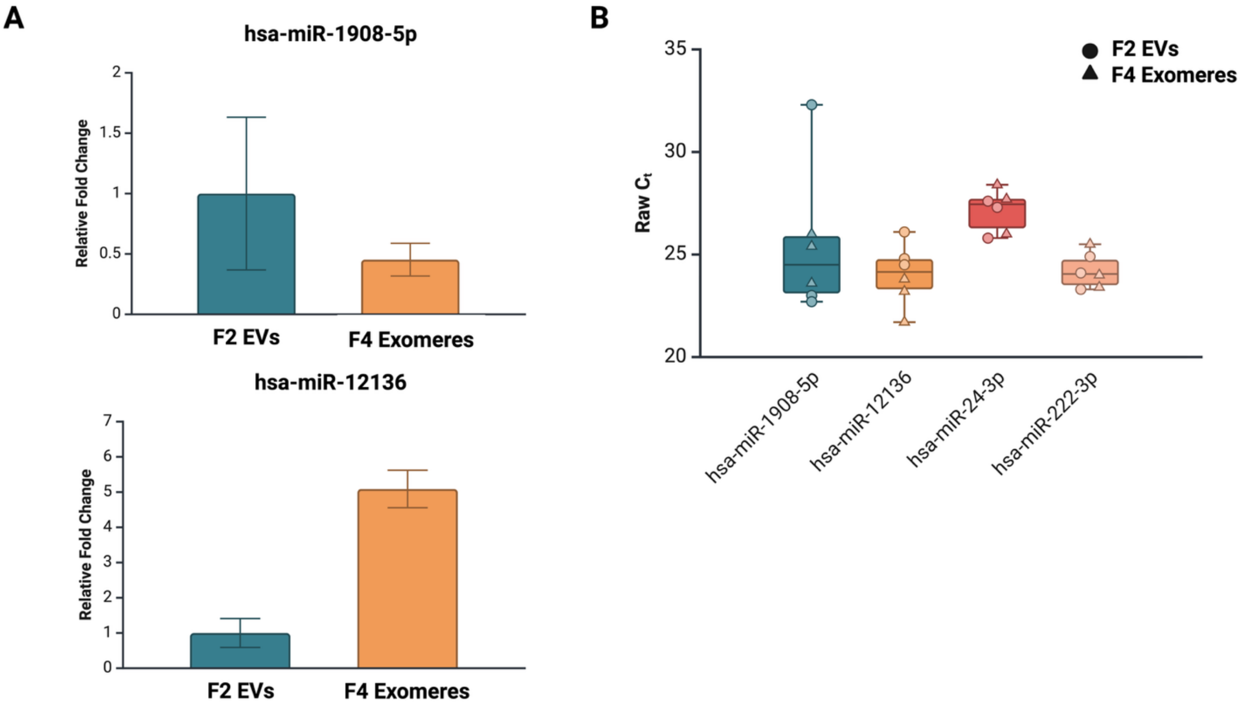

C

	Normalized Read Counts (Median of Ratios)						
**miR**	**F2 EVs - 1**	**F2 EVs - 2**	**F2 EVs - 3**	**F4 EXOM - 1**	**F4 EXOM - 2**	**F4 EXOM - 3**	**baseMean**
hsa-miR-1908-5p	7188.83	3132.79	11269.26	298.90	541.99	375.06	3801.14
hsa-miR-12136	74.93	122.28	125.30	214.87	538.92	494.58	261.81
hsa-miR-24-3p	194.82	127.17	29.48	121.24	127.38	83.20	113.88
hsa-miR-222-3p	1147.94	1261.92	978.41	1162.58	1391.40	1170.08	1185.39

Supplementary Figure 6: RT-qPCR validation of differentially and stably expressed miRNAs. **A.** Relative fold change calculated using the $\Delta\Delta C_t$ method. F2 EVs were taken as the basis. The geometric mean of hsa-miR-24-3p and hsa-miR-222-3p was used for normalization of each sample. **B.** Raw C_t values for DE miRs and putative reference miRs. **C.** Normalized mean read counts from RNA sequencing for the selected miRs. Figure created with BioRender.com.

Sample	Reference miRs C_t			Sample	DE miRs from RNA Seq C_t	
	hsa-miR-24-3p	hsa-miR-222-3p	Geo Mean C_t		hsa-miR-1908-5p	hsa-miR-12136
F2 EVs - 1	27.3	24.1	25.7	F2 EVs - 1	32.3	26.1
F2 EVs - 2	27.6	24.9	26.2	F2 EVs - 2	23.0	24.8
F2 EVs - 3	25.8	23.3	24.5	F2 EVs - 3	22.7	24.5
F4 Exomeres - 1	28.4	25.5	26.9	F4 Exomeres - 1	25.4	23.8
F4 Exomeres - 2	27.7	24.0	25.8	F4 Exomeres - 2	26.0	23.2
F4 Exomeres - 3	26.0	23.4	24.7	F4 Exomeres - 3	23.6	21.7

Sample	ΔC_t		Sample	$\Delta\Delta C_t$ (F2 EVs as basis)	
	hsa-miR-1908-5p	hsa-miR-12136		hsa-miR-1908-5p	hsa-miR-12136
F2 EVs - 1	6.6	0.4	F2 EVs - 1	6.1	0.8
F2 EVs - 2	-3.2	-1.4	F2 EVs - 2	-3.8	-1.1
F2 EVs - 3	-1.8	0.0	F2 EVs - 3	-2.4	0.3
F4 Exomeres - 1	-1.5	-3.1	F4 Exomeres - 1	-2.0	-2.8
F4 Exomeres - 2	0.2	-2.6	F4 Exomeres - 2	-0.3	-2.3
F4 Exomeres - 3	-1.1	-3.0	F4 Exomeres - 3	-1.6	-2.6

Avg. ΔC_t (F2 as basis)	0.5	-0.3

Sample	$2^{-\Delta\Delta Ct}$ (F2 EVs as basis)		Sample	Relative Fold Change	
	hsa-miR-1908-5p	hsa-miR-12136		hsa-miR-1908-5p	hsa-miR-12136
F2 EVs - 1	0.01447	0.58329	F2 EVs - 1	0.00233	0.49783
F2 EVs - 2	13.49239	2.12492	F2 EVs - 2	2.17271	1.81358
F2 EVs - 3	5.12293	0.80681	F2 EVs - 3	0.82496	0.68860
F4 Exomeres - 1	4.14042	6.88334	F4 Exomeres - 1	0.66674	5.87479
F4 Exomeres - 2	1.25052	4.77619	F4 Exomeres - 2	0.20137	4.07638
F4 Exomeres - 3	3.04107	6.22430	F4 Exomeres - 3	0.48971	5.31231

Average $2^{-\Delta\Delta Ct}$ (F2 EVs as basis)	6.20993	1.17167

Supplementary Figure 6 (continued): Relative fold change calculations for F2 EV and F4 exomere miRs validated with RT-qPCR.

References:

1. J. Ren, Z. Li and F.-S. Wong, *Journal of Membrane Science*, 2006, **279**, 558–569.
2. S. Singh, K. C. Khulbe, T. Matsuura and P. Ramamurthy, *Journal of Membrane Science*, 1998, **142**, 111–127.
